# Recovery of renal function after acute kidney injury requiring continuous renal replacement therapy

**DOI:** 10.1186/cc13579

**Published:** 2014-03-17

**Authors:** HY Jung, KH Kim, SC Park, JY Choi, SH Park, CD Kim, YL Kim, JH Cho

**Affiliations:** 1 Kyungpook National University Hospital, Daegu, South Korea

## Introduction

Acute kidney injury (AKI) is increasingly common in critically ill patients and many patients with severe kidney injury require continuous renal replacement therapy (CRRT). However, little is known regarding the incidence rate and associated factors for developing chronic kidney disease after CRRT in AKI patients. This study aimed to investigate renal outcome and the factors associated with incomplete renal recovery in AKI patients who received CRRT.

## Methods

Between January 2011 and August 2013, 397 patients received CRRT in our ICU. Among them, patients who had normal renal function before AKI and were discharged without maintenance renal replacement therapy (RRT) were included in this study. We examined the incidence of incomplete renal recovery with estimated glomerular filtration rate (eGFR) <60 ml/minute/1.73 m^2 ^during follow-up. Factors that increased risk of incomplete renal recovery after AKI were investigated with multiple logistic regression.

## Results

Forty-one AKI patients were discharged without further RRT and followed up for a mean of 7 months. Sixteen (39.0%) of 41 patients incompletely recovered their renal function. Patients with incomplete renal recovery showed older age and longer duration of anuria compared with complete renal recovery patients (69.7 ± 7.0 vs. 54.8 ± 16.9 years, *P *= 0.002; 128.6 ± 192.1 vs. 26.9 ± 66.6 hours, *P *= 019). Multivariate analysis adjusting for sex, initial eGFR, hemoglobin level, diabetes mellitus and hypertension showed that old age and long duration of anuria were independent risk factors for incomplete renal recovery (OR = 1.143, 95% Cl = 1.020 to 1.281, *P *= 0.021 and OR = 1.011, 95% Cl = 1.001 to 1.032, *P *= 0.038, respectively).

## Conclusion

The renal outcome of severe AKI requiring CRRT was poor even in patients with previous normal renal function. Long-term monitoring of renal function is needed especially in severe AKI patients with old age and long duration of anuria.

